# Efficacy of Synbiotics in Patients with Slow Transit Constipation: A Prospective Randomized Trial

**DOI:** 10.3390/nu8100605

**Published:** 2016-09-28

**Authors:** Chao Ding, Xiaolong Ge, Xueying Zhang, Hongliang Tian, Hongkan Wang, Lili Gu, Jianfeng Gong, Weiming Zhu, Ning Li

**Affiliations:** 1 Department of General Surgery, Jinling Hospital, Medical School of Nanjing University, Nanjing 210002, China; jinlingh_dc@163.com (C.D.); xiaolongge9118@126.com (X.G.); 15996282291@163.com (X.Z.); kevin_thl@163.com (H.T.); njumedgll@126.com (L.G.); jinlingh_zwm@sina.com (W.Z.); 2 First Affiliated Hospital, School of Medicine, Zhejiang University, Hangzhou 310003, China; njumedcy@126.com

**Keywords:** synbiotic, soluble dietary fiber, slow transit constipation, microbiota

## Abstract

Synbiotic intake may efficiently restore the balance of gut microbiota and improve gastrointestinal functions. The aim of the study was to evaluate the efficacy of a synbiotic in patients with slow transit constipation. A total of 100 patients with slow transit constipation were randomized to receive either a synbiotic or placebo twice daily for 12 weeks. The primary efficacy endpoints were the clinical remission and improvement rates at weeks 4 and 12. Stool frequency and consistency, colonic transit time (CTT), evacuation and abdominal symptoms, patient assessment of constipation symptoms, gastrointestinal quality-of-life index scores, satisfaction scores, and adverse events were also monitored. The clinical remission rates reached 37.5% at week 4 and 45.8% at week 12 in the treatment group, compared to 13.3% at week 4 and 16.7% at week 12 in the placebo group (*p* < 0.01 for both comparisons). Over 12 weeks, 64.6% of the patients who received the synbiotic experienced clinical improvement, compared to 29.2% of the patients in the placebo group (*p* < 0.01). During the intervention period, patients who were treated with the synbiotic exhibited increased stool frequency, improved stool consistency, decreased CTT, and improved constipation-related symptoms. This randomized, placebo-controlled trial suggested that dietary supplementation with a synbiotic improved evacuation-parameters-associated symptoms and colonic motility in patients with slow transit constipation (STC).

## 1. Introduction

Chronic constipation has become a common, often long-term, functional gastrointestinal disease that influences the quality of life in patients worldwide [1]. According to the Rome III criteria for chronic constipation [2], almost 16% of all adults are affected by chronic constipation worldwide, and it is more prevalent and symptomatic in women and elderly people [3]. Constipation is defined as difficult or infrequent passage of stool, hardness of stool, or a feeling of incomplete evacuation [4]. Clinically, constipation can always be categorized as normal transit constipation (NTC), slow transit constipation (STC), pelvic floor dysfunction, or a defecatory disorder due to assessments of anorectal function and colonic transit time [5]. Among these, STC is the major category and is characterized by a decreased rate of colonic transit [5].

The treatments for chronic constipation are varied, but remain challenging [6]. Most patients with chronic constipation have used laxatives (osmotic or stimulant) or prokinetic agents to alleviate symptoms empirically [7]. Although there is a wide range of medications, many patients are still dissatisfied with their current treatments, according to the results of a long-term survey, due to insufficient efficacy and some adverse effects [8]. Sajid et al. [9] reported that adverse events or side effects such as abdominal cramps, rash, excessive flatulence, and dizziness have occurred in constipated patients who used prucalopride, which is a new pharmacotherapy for chronic constipation. From our clinical experience in constipation, laxatives or other agents could be efficient at the beginning of chronic constipation, but they gradually become largely ineffective. Therefore, novel effective therapies are still needed.

Probiotics are live microorganisms that may benefit human health, and are now used widely to treat some diseases. Cui et al. [10] reported that *Bifidobacteria* intake could play a role in the remission of ulcerative colitis (UC) and that prebiotics, such as dietary fiber, are ingredients in food that may increase the functions of probiotics in the human body. Previous research has suggested that a sufficient intake of dietary fiber with prebiotic effects is necessary for patients with chronic constipation [11,12,13]. Pectin, one typical kind of dietary fiber, is usually present in the cell walls of fruits, vegetables, and legumes [14]. It is fermented by the intestinal microbiota in the gut and can strongly stimulate the growth and activity of some bacteria, such as *Bifidobacterium* and *Lactobacillus* [14]. Some reports have also shown that therapy with increasing dietary fiber intake, especially soluble fibers, was beneficial for individuals with chronic constipation [15]. Soluble dietary fiber, which includes pectin, is physiologically important [16]. Pectin can be digested into short-chain fatty acids (SCFAs) by intestinal microbiota, which may have effects on motility [17]. Fukumoto et al. [18] reported that SCFAs could stimulate the colon to release serotonin, which is an important factor in colonic motility. In addition, butyrate is used in treating various gastrointestinal motility disorders that are associated with the inhibition of colonic transit [17].

Currently, the combination of prebiotics and probiotics is called synbiotics, and it may have synergistic effects [19]. Morelli et al. [20] suggested that microbiota composition could be modified by synbiotics, which might play a role in gastrointestinal functions. This prospective, randomized study was designed to measure the effects of a symbiotic consisting of *Enterococci*, *Bifidobacteria*, and *Lactobacilli* triple viable bacteria (BIFICO) and pectin on slow transit constipation [10]. This was the first study to assess a specific synbiotic containing triple viable bacteria and pectin in individuals with constipation.

Our objective was to evaluate the clinical efficacy of synbiotic treatment in individuals with slow transit constipation. The primary aim was to assess clinical improvement and remission at weeks 4 and 12. The secondary aim was to assess the frequency of bowel movements, stool consistency, and colonic transit time. Other aims included the assessment of constipation-related symptoms, and the gastrointestinal quality-of-life index.

## 2. Materials and Methods

### 2.1. Ethical Issues

This study was registered in the Clinical Trials Database (ID: NCT02844426) and conducted at Jinling Hospital, a teaching hospital of Nanjing University. The current study was approved by the Ethical Committee of Jinling Hospital. All participants provided written informed consent.

### 2.2. Patients

Patients were eligible if they fulfilled the following criteria:

Inclusion criteria: age ≥18 years; body mass index 18.5–25 kg/m^2^; chronic constipation was diagnosed according to the Rome III criteria with two or fewer spontaneous, complete bowel movements (SCBMs) per week for a minimum of 6 months [21]; colonic transit time (CTT) >48 h [22]; mild-to-moderate constipation with a Wexner constipation scale score between 16 and 25 [23,24].

Exclusion criteria: Megacolon, intestinal obstruction, inflammatory bowel disease, and cancer; secondary constipation (i.e., due to drugs, endocrine disorders, neurological disorders, metabolic disorders, psychological disorders or abdominal surgery); severe anterior rectocele or full thickness rectorectal intussusception according to defecography; pregnant or lactating women; infection with an enteric pathogen; usage of antibiotics or proton pump inhibitors (PPIs); hepatic, renal, cardiovascular, respiratory or psychiatric disease; and other diseases or factors evaluated by the investigator which could influence intestinal transit or intestinal microbiota [24].

### 2.3. Study Design

A total of 100 patients were screened for eligibility to participate in our study. The sealed envelope method was used to randomize the participants into either the treatment group or the placebo group. After a week of non-interventional clinical observation, the treatment or placebo group blindly received the synbiotic or placebo twice daily for 12 weeks. The synbiotic (BIFICOPEC) contained 0.63 g of bifid triple viable capsules (BIFICO) [10] and 8 g of soluble dietary fiber (Pectin, provided by Ander Group in Yantai, China) [24]. The placebo group was treated with digestible maltodextrin (CTFH pharmaceutical company, Nanjing, China) by an experienced doctor. These constipated patients were advised to participate in a healthy lifestyle, including proper diet and exercise, and to avoid any other probiotics and dietary fiber during the study period. If patients did not have a bowel movement for 3 or more consecutive days, they were permitted to take up to 20 g of Macrogol 4000 powder (Forlax^®^, Ipsen, Paris, France). If ineffective, an enema could be used.

During the follow-up, patients were asked to keep daily diaries of their bowel symptoms, including stool consistency, as rated by the Bristol Stool Form Scale (BSFS). The trained physicians, who were blinded to the treatments, assessed the quality of life and constipation-related symptoms of all of the participants at weeks 4 and 12 via phone or e-mail. Adverse events were also monitored during follow-up.

### 2.4. Outcomes

The primary efficacy endpoints were as follows: (1) Clinical remission rate: the proportion of patients having an average of three or more spontaneous complete bowel movements (SCBMs) per week during the observation period of weeks 4 and 12; and (2) Clinical improvement rate: the proportion of patients with an average increase of one or more SCBMs per week compared with baseline at weeks 4 and 12.

The secondary efficacy endpoints were as follows: (1) Number of bowel movements within one week [24]; (2) Stool consistency according to the BSFS: stool types 1 and 2 indicated constipation, types 3, 4, and 5 indicated a normal consistency, and types 6 and 7 indicated diarrhea [24]; and (3) Colonic transit time (CTT), which was measured at baseline and at weeks 4 and 12 by the Metcalf method [22].

Other endpoints included the following: (1) The Patient Assessment of Constipation Symptoms (PAC-SYM) questionnaire was administered at baseline and at weeks 4 and 12. The questionnaire contained 12 symptoms that were grouped into three subscales for stool, abdominal, and rectal symptoms. For the overall scale and each subscale, the scores ranged from 0 (symptoms absent) to 4 (symptoms very severe) [25]; (2) The Gastrointestinal Quality-of-Life Index (GIQLI) assessment, which was used to evaluate the quality of life in patients with gastrointestinal diseases, comprised 36 questions using a 5-point Likert-type scale ranging from 0 to 4 (0, worst; 4, best) [26]; (3) The satisfaction scores of constipated patients, which used a 5-point ordinal scale. The score ranged from 1 (extremely unsatisfied) to 5 (extremely satisfied); (4) For evacuation symptoms, patients recorded their perception of straining, lumpy hard stools, the sensation of incomplete evacuation, and the sensation of anorectal blockage according to a 5-point ordinal scale (1, none; 2, mild; 3, moderate; 4, severe; or 5, very severe); (5) Finally, abdominal symptoms were categorized, patients recorded their symptoms of abdominal pain or cramps and bloating or flatulence according to five classifications (1, none; 2, mild; 3, moderate; 4, severe; or 5, very severe).

### 2.5. Safety Assessments

During treatment and follow-up, patients were advised to record adverse events in daily diaries and to report adverse events immediately. Adverse events could include abdominal pain, flatulence, borborygmus, and other gastrointestinal symptoms.

### 2.6. Sample Size

The sample size was calculated based on the frequency of evacuation and the standard deviation of the difference as 0.8 between the groups [27]. Therefore, a total sample size of 100 (50 in each group) was sufficient to expect a 95% power with a two-sided significance level of 0.05.

### 2.7. Statistical Analysis

The results were analyzed with SPSS 19.0 (SPSS, Inc., Chicago, IL, USA). Continuous data were presented as the mean ± standard deviation and categorical data were presented as *n* (%). Paired *t* tests or a repeated measures ANOVA were performed for continuous variables, and for categorical variables; Pearson’s chi-square test or the Fisher exact test was performed as appropriate. *p* values < 0.05 were considered statistically significant for all comparisons.

## 3. Results

### 3.1. Baseline Characteristics

**Figure 1:**
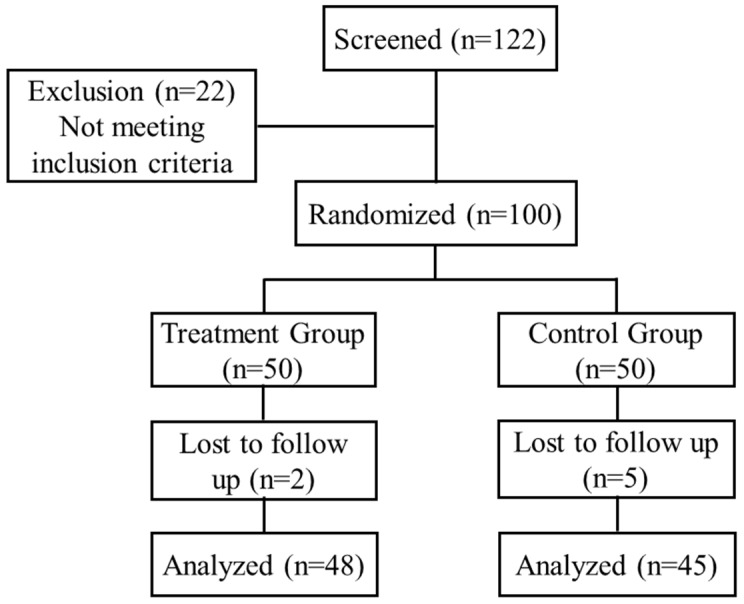
Consolidated standards of reporting trials (CONSORT) flow diagram of patients recruitment and analysis.

In our study, a total of 100 patients were enrolled and randomized into two groups, with 50 participants per group. Seven patients did not complete the study protocol. Therefore, a total of 93 patients, including 48 patients who had received the synbiotic and 45 patients who had received placebo, were included in the final analysis. The patient flow is detailed in Figure 1. The baseline characteristics of patients in the treatment or placebo group are shown in Table 1. Most enrolled patients were females (63.44%) compared to males (36.56%). The disease durations of 7.1 ± 4.2 years and 7.4 ± 3.9 years in the placebo and treatment groups, respectively, were not significantly different. There were also no differences in gender, age, BMI, Wexner score, stool consistency, or colonic transit time.

### 3.2. Primary and Secondary Efficacy Endpoints

During the follow-up period, more patients in the synbiotic group achieved a mean of three or more bowel movements per week than in the placebo group at both weeks 4 and 12, and the clinical remission rate in the synbiotic group reached 18% at week 4 and 22% at week 12. There were significant differences in clinical improvement in the synbiotic and the placebo groups at weeks 4 and 12 (*p* < 0.01). After treatment, compared to the placebo group, the number of bowel movements in the synbiotic group improved significantly within one week and reached 4.5 ± 1.6 and 5.1 ± 2.0 at weeks 4 and 12 (*p* < 0.001). In addition, the stool consistency score was statistically significantly increased in the treatment group compared to the placebo group (week 4, 3.2 ± 1.2 vs. 2.5 ± 0.8, *p* < 0.001; week 12, 3.5 ± 1.1 vs. 2.4 ± 0.8, *p* < 0.001). The results of CTT showed that patients who had received the synbiotic treatment had a shorter transit time than did patients in the placebo group at weeks 4 and 12, which could reflect improved intestinal motility. The detailed data are shown in Table 2.

### 3.3. Other Efficacy Results

Treatment with the synbiotic relieved the symptoms of constipated patients. Compared with baseline, the PAC-SYM score significantly decreased in the treatment group at weeks 4 and 12 (*p* < 0.001). However, there was no statistically significant decrease in the PAC-SYM score in the placebo group (Table 3). As shown in Table 3, the GIQLI score in the synbiotic group was 83.5 ± 12.6 before treatment, and it increased to 117.8 ± 15.8 at week 4 (*p* < 0.01) and 126.9 ± 16.5 at week 12 (*p* < 0.001). Although the GIQLI score in the placebo group also improved from 86.3 ± 11.2 to 91.7 ± 12.8 at week 4 and 95.5 ± 15.3 at week 12, no significant difference was found. We also recorded the satisfaction scores of constipated patients during follow-up. Similarly, satisfaction scores in the placebo and treatment groups were analyzed. Our results showed that the score in the treatment group was significantly higher than in the placebo group, not only at week 4, but also at week 12—which indicates that more patients were satisfied with the treatment (Table 3).

**Figure 2:**
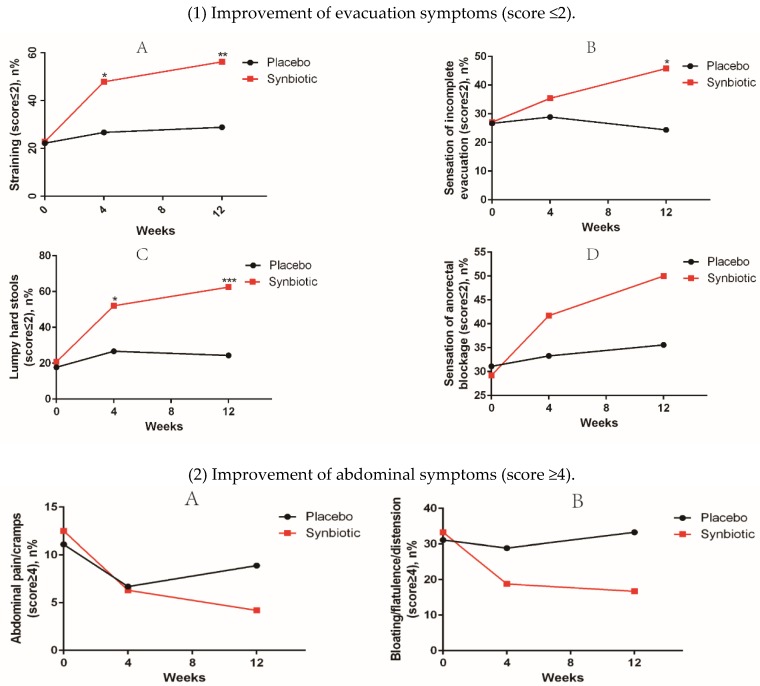
Improvement of constipation-ralated symptoms in the treatment vs. placebo groups. 5-point ordinal scale, 1 indicates none, 2 mild, 3 moderate, 4 severe, and 5 very severe. * *p* value < 0.05; ** *p* value < 0.01; *** *p* value < 0.001. (1) Improvement of evacuation symptoms in treatment vs. placebo groups at baseline, week 4, and week 12; (2) Improvement of abdominal symptoms in treatment vs. placebo groups at baseline, week 4, and week 12.

In terms of evacuation symptoms, patients in the treatment group reported significantly less straining and fewer lumpy hard stools (Figure 2). No significant improvement was found in the sensation of incomplete evacuation at week 4, but a significantly higher proportion of patients in the treatment group at week 12 reported an improvement in this sensation (*p* < 0.05) (Figure 2). However, there was no significant difference in the sensation of anorectal blockage between groups (Figure 2). For abdominal symptoms, a trend toward improvement was found in the treatment group, but there were no significant differences between the groups (Figure 2). No significant adverse events associated with treatment were reported.

## 4. Discussion

This was a prospective randomized trial to evaluate the efficacy of a synbiotic (BIFICOPEC) comprised of probiotics (BIFICO) [10] and soluble dietary fiber (Pectin) in patients with slow transit constipation who met the Rome III criteria. Our study found that 12 weeks of supplementation with probiotics and soluble dietary fiber increased bowel movements, improved the PAC-SYM and GIQLI scores, relieved constipation-related symptoms, and decreased CTT. Finally, the clinical remission and clinical improvement rates reached 45.8% and 64.6%, respectively, at week 12 among patients with mild-to-moderate constipation. No serious treatment-related adverse events were observed during the follow-up period.

Slow transit constipation, which is one type of chronic idiopathic constipation, has an important pathophysiological feature of decreased colonic motility, which could be diagnosed by using radiopaque markers, as occurred in this study [28]. Recently, many researchers have focused on the relationship between intestinal microbiota and constipation and have demonstrated that intestinal microbiota contribute to the pathophysiology of functional gastrointestinal disorders [29]. Parthasarathy et al. [30] suggested that the profile of microbiota in the intestine was associated with colonic transit, and genera from *Firmicutes* was related with faster colonic transit. Zhu et al. [31] reported that, compared with a control group, individuals in a constipation group had a distinct microbiome in the gut. Moreover, the Bristol Stool Scale classification has been widely used to reflect intestinal colon transit time in constipated patients. Vandeputte et al. [32] advised that stool consistency, as evaluated using the Bristol Stool Scale, was strongly correlated with intestinal microbiota. These studies all suggest that gut microbiota contribute to the etiology in constipation. Our previous study [33] reported that the reestablishment of the whole intestinal flora with fecal microbiota transplantation could alleviate the symptoms of slow transit constipation, which might provide a basis for the considerable role of gut microbiota in constipation from the perspective of clinical treatment.

Recently, emerging studies on the individual benefits of probiotics and prebiotics in the treatment of chronic constipation have been reported. Ford et al. [19] reported a systematic review and meta-analysis and showed that probiotics appeared to have beneficial effects in chronic idiopathic constipation (CIC), but only a few RCTs were available for the analysis. Data from RCTs for prebiotics and synbiotics in individuals with CIC are also sparse. Some studies found that prebiotics had a positive effect in constipated patients. Christodoulides et al. [34] suggested that fiber was moderately effective for chronic idiopathic constipation in adults. Suares et al. [35] also reported that soluble fiber might be more beneficial than insoluble fiber in constipated patients in alleviating straining, pain on defecation, improving stool consistency, and other constipation-related symptoms. In addition, research on the changes in intestinal microbiota in individuals with constipation indicated that microbiota other than *Lactobacillus* and *Bifidobacteria* also changed, which might indicate the importance of stability and integration of intestinal microbiota. Therefore, supplementation with both probiotics and prebiotics is better than treatment with probiotics or prebiotics alone.

Pectin is an important soluble dietary fiber that can be fermented by gut microbiota. The most familiar and predominant structural element in pectin is formed by the “smooth” homogalacturonan regions and is composed predominantly of a homopolymer of partially methyl esterified (1–4)-linked α-d-galacturonic acid (GalA) units [12]. The health benefits of pectin might include alterations in the composition of intestinal microbiota and the production of short-chain fatty acids [36,37]. Onumpai et al. [37] reported that pectin could stimulate the activity of *Bifidobacterium* and *Lactobacillus*. Recently, our team also found that fecal microbiota transplantation, in combination with soluble dietary fiber, could improve the symptoms of patients with slow transit constipation, indicating that the regulation of intestinal microecology was associated with constipation [24]. The addition of pectin to the treatment regimen further improved the symptoms in constipated patients. Therefore, pectin may play a beneficial role in constipation.

In contrast, butyrate, which is a byproduct of pectin fermentation by certain microbiota, is necessary for colonic homeostasis and provides energy for intestinal epithelial cells [38]. We chose maltodextrin as a placebo control because maltodextrin is an easily digested carbohydrate, but it is not fermented by intestinal microbiota; thus, it would not affect gut metabolism and microbial ecology [27].

In our study, we provided constipated patients with bifid triple viable capsules and pectin for 12 weeks. Our results revealed an improvement in the number of bowel movements per week, stool consistency, and colonic transit time in constipated patients, which are related to intestinal motility. Intestinal microbiota analysis has already shown that microbiota are associated with colonic transit, stool frequency, and stool consistency in humans [30,32]. During the follow-up, we found that the improvement in stool consistency was the most obvious effect, but a significant improvement in the sensation of incomplete evacuation did not appear until three months after treatment initiation. Harder stools and decreased frequency are associated with a slower colonic transit time, while increased incomplete evacuation is related to outlet obstructive constipation [22]. So the efficacy of synbiotics in constipation might depend on the improvement of intestinal motility through regulating intestinal microecology.

Because pharmacological interventions have limited efficacy and more side effects, traditional treatments have not been able to fully satisfy constipated patients [39]. Prucalopride is a widely used prokinetic agent, but adverse events such as abdominal cramps, headache, skin disorders, and drug dependence have been reported with its use [9]. During our treatment and follow-up, no serious adverse events occurred in the patients. It is found that constipated patients at our hospital prefer to use prebiotics, probiotics, or synbiotics rather than some laxatives.

However, our pilot study does have several limitations. First, this was a single-center study, and the sample size of our trial was relatively small. A multicenter randomized controlled study should be performed to verify these findings. Second, our follow-up period was restricted to 12 weeks. The clinical efficacy of treatment for chronic constipation should be examined in a study with a longer follow-up. Finally, we did not analyze the structural changes in the gut microbiota in constipated patients before and after treatment. Intestinal microbiota analysis might provide us a new perspective to explain the therapeutic mechanism underlying synbiotic treatment.

## 5. Conclusions

In conclusion, we found that 12 weeks of treatment with a synbiotic that contained pectin as a prebiotic and bifid triple viable capsule (BIFICO) as a probiotic was effective in increasing stool frequency, improving stool consistency, decreasing colonic transit time, and relieving constipation-related symptoms. In addition, synbiotic treatment effectively improved the quality of life in patients with mild-to-moderate constipation. Therefore, additional multicenter randomized clinical trials are needed to confirm these results and assess the role of the regulation of gut microbiota in treatment of constipation.

## Figures and Tables

**Table 1:** Baseline demographics in patients received treatment or placebo.

Characteristics	Placebo (*n* = 45)	Treatment (*n* = 48)	*p* Value
Sex (male/female) *	16 (35.6)/29 (64.4)	18 (37.5)/30 (62.5)	0.846
Age (year) ^†^	48.3 ± 11.3	47.2 ± 10.7	0.638
BMI (kg/m^2^) ^†^	22.8 ± 1.1	22.6 ± 1.1	0.305
Disease duration (year) ^†^	7.1 ± 4.2	7.4 ± 3.9	0.695
Wexner score ^†^	19.8 ± 2.0	20.0 ± 2.2	0.797
No. of BMs/week ^†^	2.1 ± 0.6	2.2 ± 0.7	0.615
Stool consistency ^†^	2.0 ± 0.6	2.1 ± 0.5	0.366
CTT (h) ^†^	73.0 ± 10.3	71.7 ± 10.8	0.567
Smoker *	3 (6.7)	4 (8.3)	0.761
Alcohol consumer *	6 (13.3)	5 (10.4)	0.663
Regular exercise *	12 (26.7)	14 (29.2)	0.788

BMI, body mass index; BM, bowel movement; CTT, colonic transit time. * Values are expressed as *n* (%), ^†^ values are expressed as the mean ± standard deviation.

**Table 2:** Clinical outcomes of treatment vs. placebo groups.

Endpoint	4 Week		12 Week	
	Placebo	Synbiotic	Placebo	Synbiotic
Clinical remission rate (%) ^†^	6 (13.3)	18 (37.5) **	8 (16.7)	22 (45.8) **
Clinical improvement rate (%) ^†^	11 (24.4)	25 (52.1) **	14 (29.2)	31 (64.6) **
No. of BMs/week ^‡^	2.9 ± 1.1	4.5 ± 1.6 ***	3.1 ± 1.4	5.1 ± 2.0 ***
Stool consistency ^‡^	2.5 ± 0.8	3.2 ± 1.2 ***	2.4 ± 0.8	3.5 ± 1.1 ***
CTT (h) ^‡^	68.2 ± 11.3	53.8 ± 10.9 **	70.5 ± 12.1	49.3 ± 11.7 ***

BM, bowel movement; CTT, colonic transit time. ^†^ Values are expressed as *n* (%), ^‡^ values are expressed as the mean ± SD. ** *p* value < 0.01; *** *p* value < 0.001.

**Table 3:** Efficacy endpoints.

Week	PAC-SYM ^†^		GIQLI ^†^		Satisfaction Score ^†^	
	Placebo	Synbiotic	Placebo	Synbiotic	Placebo	Synbiotic
Baseline	1.9 ± 0.3	1.9 ± 0.2	86.3 ± 11.2	83.5 ± 12.6	—	—
4 weeks	1.8 ± 0.3	1.4 ± 0.5 **	91.7 ± 12.8	117.8 ± 15.8 **	2.8 ± 1.2	3.5 ± 1.4 *
12 weeks	1.7 ± 0.4	1.2 ± 0.6 ***	95.5 ± 15.3	126.9 ± 16.5 ***	2.9 ± 1.3	3.8 ± 1.4 **

GIQLI, Gastrointestinal Quality-of-Life Index; PAC-SYM, Patient Assessment of Constipation Symptoms. ^†^ Values are expressed as the mean ± SD; — indicates not applicable. * *p* value < 0.05; ** *p* value < 0.01; *** *p* value < 0.001.
